# Feasibility of climate reanalysis data as a proxy for onsite weather measurements in outdoor thermal comfort surveys

**DOI:** 10.1007/s00704-022-04129-x

**Published:** 2022-07-07

**Authors:** Eduardo L. Krüger, Claudia Di Napoli

**Affiliations:** 1 Universidade Tecnológica Federal do Paraná, Curitiba, Brazil; 2 School of Agriculture, Policy and Development, University of Reading, Reading, UK; 3 Department of Geography and Environmental Science, University of Reading, Reading, UK

**Keywords:** Climate reanalysis, Outdoor thermal comfort, Questionnaire survey, UTCI, Dynamic Thermal Sensation, ERA5-HEAT

## Abstract

Outdoor thermal comfort (OTC) surveys require synchronous monitoring of meteorological variables for direct comparisons against subjective thermal perception. The Universal Thermal Climate Index (UTCI) is a feasible index as it integrates meteorological conditions as a single value irrespective of urban morphological attributes or biological sex, age and body mass. ERA5-HEAT (Human thErmAl comforT) is a downloadable reanalysis dataset providing hourly grids of UTCI climate records at 0.25° × 0.25° spatial resolution from 1979 to present. We here evaluate for the first time whether it is possible to use ERA5-HEAT data as a proxy for the UTCI measured onsite during OTC surveys. A dataset comprising 1640 survey responses gathered over 14 OTC campaigns in Curitiba, Brazil (25°26′S, 49°16′W) was analysed. We assessed the bias obtained between the Dynamic Thermal Sensation, an index derived from the UTCI, and the thermal sensation reported by survey participants by considering locally measured meteorological variables and ERA5-HEAT reanalysis data. As ERA5-HEAT data are given on an hourly basis, prediction bias can be greatly reduced when accounting for survey responses close to the hour. In terms of seasons, the fall and winter seasons have diminished mean bias, though with larger spread than in summer. In terms of UTCI stress categories, prediction bias is lower for the thermal comfort range. When comparing reanalysis data against WMO station data as proxy candidates for survey field data, the former presented lower bias, less spread in terms of standard deviation and higher correlation to *in situ* data.

## Introduction 

Outdoor thermal comfort (OTC) is likely to become an increasingly important area of research as climate change mitigation and pandemic resiliency in urban centres demand more and more for climate-responsive urban planning. The way OTC is scrutinized in research is based on a human-centred approach that looks at pedestrians’ thermal perception and establishes linkages to microclimatic conditions throughout right-here-right-now questionnaire surveys. The survey method for evaluating thermal conditions has been employed in indoor spaces first. Fanger and colleagues adopted it in the 1970s in climate-chamber studies that later became the basis for the development of the predicted mean vote ‘PMV’ index (Fanger 1970). The comparison between *subjective* personal thermal responses and *objective* microclimatic conditions allows researchers in the field to establish thermal comfort ranges. These can ultimately be used as target conditions in different thermal environments. The very definition of thermal comfort presented by ANSI/ASHRAE (2004) involves the subjective response (the ‘condition of mind’) that is to be assessed in a given thermal environment through subjective evaluation.

In outdoor spaces, this human-centred approach has been used since the late 1990s and was crucial for evaluating the goodness-of-it (or lack thereof) of existing comfort models that were originally developed for indoor spaces. Discrepancies between the static comfort models developed for indoor spaces and the dynamic, transient conditions presented by outdoor spaces were found thus unveiling the need for more adequate comfort models (Lau et al. 2019). The Universal Thermal Climate Index (UTCI)-Fiala model could address this need. One of its goals was to create an index, the UTCI, able to assess the dynamic physiological response of humans interacting with microclimatic conditions (Fiala et al. 2003). The index was developed and implemented within the framework of the International Society of Biometeorology (ISB), from initial discussions that took place during the International Congress of Biometeorology in Sydney, Australia, in 1999 and were actualized in the COST Action 730 (https://www.cost.eu/actions/730/).

Since its launch in 2009 (when the COST Action 730 was completed), the UTCI has been applied in multiple research fields resulting in over 300 peer-reviewed articles, with about one-third of these related to OTC and thermal stress in outdoor environments (Krüger 2021). Moreover, the index has been recently used by the City of London in their Thermal Comfort Guidelines (https://www.cityoflondon.gov.uk/assets/Services-Environment/thermal-comfort-guidelines-for-developments-in-the-city-of-london.pdf), which are part of the British planning system for assessing the impact of new developments on urban microclimate, including streets, parks, public gardens and spaces. The UTCI is to be included as a feasible index for evaluating human biometeorology in cities also in one of the guidelines of the Association of German Engineers (Verein Deutscher Ingenieure ‘VDI’).

Applications of the UTCI related to OTC have been using questionnaire-based surveys with pedestrians and users of open-air spaces (Krüger 2021). During surveys, microclimatic conditions, which are defined by environmental variables, such as air temperature, humidity, wind speed and mean radiant temperature, are measured and monitored using instruments such as portable weather stations. These offer the advantage of recording environmental variables at high spatial and temporal resolution, i.e. at the same time and location at which questionnaire surveys are carried out. However, the possibility of missing survey data due to mishaps in the recording or damages in the monitoring equipment cannot be fully dismissed. Data from nearby stationary meteorological stations may also present gaps in their records, so that subjective thermal responses data may end up having no meteorological counterparts. Furthermore, standardized and agreed protocols for the onsite measurement of environmental variables (mean radiant temperature in particular) are currently lacking (Johansson et al. 2014). This makes the inter-comparison of outcomes from individual OTC surveys rather problematic.

As a comprehensive description of the observed climate as it has evolved during recent decades, a climate reanalysis has the potential to overcome these issues. A climate reanalysis uses data assimilation techniques developed for weather forecasting to combine historical observations from meteorological stations, as well as ships, balloons and satellites, with outputs from numerical weather prediction (NWP) models. The result is a consistent description of the atmosphere-land–ocean system in the form of maps without gaps, i.e. regular longitude/latitude grids at consequent time steps spanning the recent past. One climate reanalysis is ERA5, which is produced by the European Centre for Medium-Range Weather Forecasts (ECMWF) within the Copernicus Climate Change Service (C3S) (Hersbach et al. 2020). ERA5 provides land and atmospheric parameters since 1950 on world-wide hourly-stepped grids at 0.25° × 0.25° spatial resolution (approximately 31 × 31 km), which is the highest possible resolution to date for a global climate reanalysis. Among the parameters provided are 2 m air temperature, 2 m dewpoint temperature, 10 m wind speed, solar and thermal radiation fluxes. Based on these, the ERA5-HEAT (Human thErmAl comforT) reanalysis dataset has been produced (Di Napoli et al., 2021a). ERA5-HEAT provides a complete historical reconstruction of the UTCI from 1979 to present as hourly gridded data covering the globe at the same resolution as ERA5 (31 × 31 km).

UTCI reanalysis data as provided by ERA5-HEAT have recently been explored in the context of OTC in urban areas. The effect of pedestrians’ history on their thermal comfort, for instance, has been assessed in two different locations in southern Brazil by retrieving ERA5-HEAT UTCI at the days, weeks and months preceding OTC surveys (Bröde et al. 2021). As pre-survey UTCI data are not available from portable weather stations (the latter measure outdoor environmental variables only for the period when OTC surveys are taken), ERA5-HEAT turned out as a relevant source for obtaining historical UTCI data. These have then been used as representative of pre-survey thermal conditions, thus allowing the evaluation of short- and long-term acclimatisation on subjective responses. For the same and other locations in Brazil, ERA5-HEAT UTCI has also been deployed for investigating adaptation to regional bioclimatic conditions by local populations (Krüger et al. 2021).

Given the increasing number of its applications in the field, a quantitative assessment of ERA5-HEAT in the context of OTC surveys is needed. The aim of the present study is therefore to evaluate whether ERA5-HEAT can be used as a proxy source for UTCI data measured on site during OTC surveys. To achieve this, thermal comfort responses from questionnaire-based surveys taken in Curitiba, Brazil, were compared against outdoor thermal conditions as expressed by the UTCI, with the latter both calculated from onsite measurements and extracted from ERA5-HEAT.

## Data and methods

In this study, a dataset consisting of 1640 survey responses gathered in 2009 over 14 OTC campaigns in Curitiba, Brazil, was used. The UTCI was calculated from onsite monitored observations using Bioklima 2.6 (Błażejczyk & Błażejczyk 2010) as well as retrieved from ERA5-HEAT via the Copernicus Data Store.

### Study area

The study was conducted at the city of Curitiba, which is located in southern Brazil. Table 1 summarizes annual temperature ranges as well as the total number of days with air temperatures below 10 °C and above 25 °C in the study area. Data are from the official weather station belonging to the Brazilian National Meteorological Service (Instituto Nacional de Meteorologia, INMET) network and refer the climatological 30-year period from 1981 to 2010.

**Table 1:** Climatic characteristics of Curitiba (https://http://www.inmet.gov.br)

Weather station	Latitude (degrees)	Longitude (degrees)	Elevation (m a.s.l.)	Annual temperature range (monthly means)	Number of days with *T* < 10 °C	Number of days with *T* > 25 °C
83842	- 25.43	- 49.27	924	13.5–21.0 °C	74	156

According to the Köppen-Geiger classification, Curitiba has an oceanic climate (Cfb, Kottek et al. 2006) with mean temperatures ranging between 20.1 and 21.0 °C in summer and between 13.5 and 14.6 °C in winter, for a mean year temperature equal to 17 °C circa.

### OTC surveys

Questionnaire-based surveys were carried out in daytime, i.e., between 10 a.m. and 3 p.m., from summer to winter 2009 (January-August) in 12 locations of Curitiba’s city centre. To record environmental variables at the time of the surveys in each location, two factory-calibrated HOBO weather stations (Onset Computer), equipped with a three-cup anemometer positioned at approximately 2.1 m from the ground (wind data were later scaled up at 10-m height as requested for the calculation of the UTCI), were used, synchronously recording field data at two different spots of the downtown area of Curitiba, per campaign (a side project looked at differences in microclimatic data due to urban morphological attributes — Krüger et al. 2011). The portable stations were also equipped with air temperature and relative humidity sensors as well as a copper, gray-painted globe thermometer with a diameter of 50 mm placed at 1.1 m. For sake of clarity, we refer to the measurements of environmental variables by the portable weather stations as *in situ* data hereafter. To record pedestrians’ OTC, thermal sensations were evaluated as reported for representative demographics (sex, age) and in a diversity of urban settings (monitoring points were located pairwise in street canyons, squares and crossroads in pedestrianized streets in the downtown area) within Curitiba’s city centre. The thermal comfort questionnaire was designed according to the recommendations of ISO 10551 (1995). Specifically, inclusion criteria were a minimum residency in Curitiba of 6 months, exposure to the outdoor environment for at least 15 min prior to the interview and healthy subjects. For the self-reported thermal sensation assessment, we employed a 7-point scale with neutral midpoint (− 3 for cold, − 2 for cool, − 1 for slightly cool, 0 for neutral, + 1 for slightly warm, + 2 for warm and + 3 for hot). A more complete description of the survey protocol is given by Rossi (2012) and Krüger et al. (2011). For this paper, the survey dataset was composed of 1640 thermal comfort responses.

### Reanalysis data

**Fig. 1:**
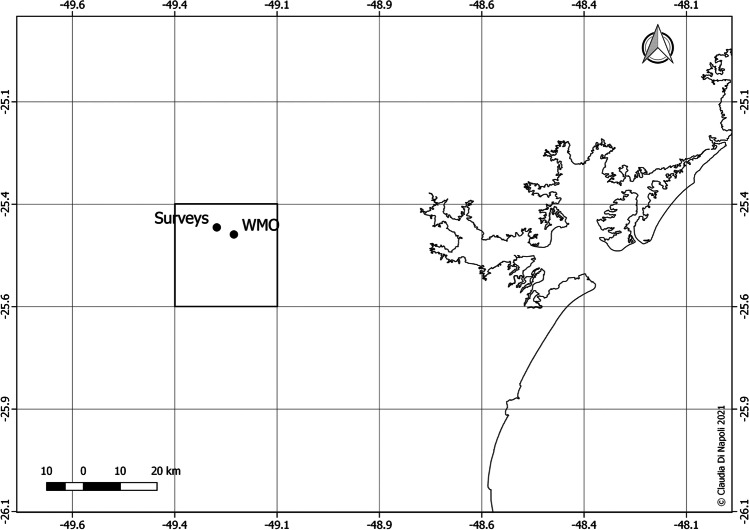
Locations of the survey area (‘Surveys’) and of the stationary WMO station (‘WMO’). The grid cell from which ERA5-HEAT UTCI data were extracted is highlighted by thicker borders

Reanalysis data from ERA5-HEAT were used for comparison against *in situ* data from OTC surveys. A gridded UTCI dataset was first retrieved from ERA5-HEAT for the January–August 2009 period via the Copernicus Climate Data Store (CDS 2020). UTCI reanalysis data at Curitiba were then extracted from the grid cell where the survey area is located (Fig. 1). It is worth noting that the survey area is one of the 12 locations surveyed in Curitiba’s downtown area and that we adopt here a midpoint reference (25°26′S, 49°16′W, 924 m a.s.l.). The extraction generated a time series of hourly UTCI reanalysis data to be compared with *in situ* UTCI data.

### WMO station data

Measurements from the local World Meteorological Organisation (WMO) weather station, which is situated close to Curitiba (25°27′S, 49°14′W, 923 m a.s.l), were also considered in the study. The WMO weather station (A807) falls in the same grid cell as the survey area (Fig. 1).

### Calculation of the UTCI and DTS

The UTCI is a multi-node thermal index based on the principle of an equivalent air temperature obtained at a given reference environment, as used by other thermal indices, most prominently by the PET (physiological equivalent temperature) index which is also widely applied in outdoor comfort research.

For the *in situ* dataset, the UTCI was obtained from the measurements of four environmental variables — air temperature, humidity, wind speed and radiation — made by the portable weather station. Wind speed was scaled up to the required height of 10 m above ground (Bröde et al. 2012a) and the mean radiant temperature (${T}_{MRT}$) was calculated by the forced convection equation as in ISO 7726 (1998). Bioklima 2.6 (Błażejczyk & Błażejczyk 2010) was then used to calculate the UTCI.

For the WMO station dataset, UTCI and ${T}_{MRT}$ were both calculated from available observations using the RayMan model (Matzarakis et al. 2007). The mean radiant temperature was determined according to one of the methods presented by Krüger et al. (2014) for estimating this variable in RayMan. The method takes in input the air temperature and humidity, wind speed and global solar radiation as measured by a weather station, and it calculates the mean radiant temperature according to the German VDI-Guideline 3786, Part 2 ‘Methods for the human-biometeorological evaluation of climate and air quality for urban and regional planning at regional level, Part I: Climate’ (VDI 1998). In the case of the WMO station, which must be located away from trees, buildings, walls or other obstructions to comply with WMO standards (WMO 2018), the method assumes the sky view factor (SVF) to be 1, which corresponds to an open field. With this assumption, the major difficulty found in applications in urban and regional planning when quantifying the shading of direct and diffuse radiation by building structures was circumvented (Matzarakis et al. 2000). In RayMan, the UTCI is estimated via a regression equation based on a heat transfer model (Fiala et al. 2012; Fröhlich et al. 2019) which accounts for the aforementioned meteorological variables as measured at the WMO station.

In ERA5-HEAT, the UTCI is determined using the operational procedure by Bröde et al. (2012b). The procedure calculates the UTCI from four climate variables, namely 2 m air temperature, 10 m wind speed, relative humidity and ${T}_{MRT}$. The ${T}_{MRT}$ is computed from solar and thermal radiation fluxes as described by Di Napoli et al. (2020). Briefly, the downwelling thermal component from the atmosphere (${L}_{surf}^{dn}$), the upwelling thermal component from the ground (${L}_{surf}^{up}$), the direct component from the sun (${I}^{*}$) and a diffuse solar component, with the latter equal to the sum of the isotropic diffuse solar radiation flux (${S}_{surf}^{dn, {{diffuse}}}$) and the surface-reflected solar radiation flux (${S}_{surf}^{up}$), are first computed from the solar and thermal data stored in the ERA5 reanalysis dataset. Then, they are input into the following formula to calculate ${T}_{MRT}$ (Staiger and Matzarakis, 2010):

$$MRT= {\left\{\frac{1}{\sigma }\left[{f}_{a}{L}_{surf}^{dn}+{f}_{a}{L}_{surf}^{up}+\frac{{\alpha }_{ir}}{{\varepsilon }_{p}}\left({f}_{a}{S}_{surf}^{dn, {{diffuse}}}+{f}_{a}{S}_{surf}^{up}+{f}_{p}{I}^{*}\right)\right]\right\}}^{0.25}$$

where $\sigma$ is the Stefan-Boltzmann constant (5.67 × 10^−8^ W/m^2^ K^4^), ${\alpha }_{ir}$ is the absorption coefficient of the body surface area irradiated by solar radiation (standard value 0.7), ${\varepsilon }_{p}$ is the emissivity of the clothed human body (standard value 0.97), ${f}_{a}$ is an angle factor, and ${f}_{p}$ is the surface projection factor. The factor ${f}_{a}$ is set to 0.5 which corresponds to considering the surroundings of a human body as made of a lower hemisphere (ground) and an upper hemisphere (sky) only (Kántor and Unger 2011). The factor ${f}_{p}$ represents the portion of body surface exposed to direct solar radiation, and, for a rotationally symmetric standing or walking person, it is computed from the solar elevation angle (Jendritzky et al. 1990). The computation of ${T}_{MRT}$ and UTCI from ERA5 climate variables is performed via an automated routine which delivers the corresponding gridded data as finished products on the Copernicus Data Store (Di Napoli et al., 2021a).

For the UTCI calculated in the three datasets, the dynamic thermal sensation (DTS) was derived. As stated by Fiala (1998), the DTS is an index for judging thermal situations. It translates the UTCI into predicted thermal sensation votes that can be more directly interpreted in terms of thermal comfort/stress categories. Specifically, we transformed UTCI values from ERA5-HEAT and station observations into DTS data via the UTCI-to-DTS conversion table by Bröde et al. (2012b). The DTS is given according to the same 7-point scale (and respective categories) as in the reported thermal votes.

### Data analysis

A comparison between *in situ* versus ERA5-HEAT data was carried out for the UTCI, ${T}_{MRT}$ and the thermal sensations reported in relation to corresponding UTCI and DTS values. The comparison was performed by means of statistical analysis. This consisted in the calculation of the following evaluation metrics: mean bias (reanalysis UTCI minus onsite UTCI), standard deviation, Pearson’s *r*-value, the *p*-value and the maximum positive and negative offsets between both, the latter serving as feasible indicators of under- and over-estimation of ERA5-HEAT reanalysis data.

As the aim of the paper is to evaluate the possibility of using reanalysis UTCI data as a proxy for missing field data (and not as a full replacement of the latter), it is interesting to evaluate under which conditions reanalysis data would yield negligible differences to field data. For that, the influence of different seasons (summer, fall, winter), urban morphological attributes of the monitoring points (street canyons, crossroads, squares) and UTCI thermal stress categories on such a relationship was evaluated as described below.

The influence of seasons was assessed by means of two-factor analysis of variance (two-way ANOVA). Such analysis allowed us to investigate the influence of categorical independent variables (the three seasons covered in the surveys) on one continuous dependent variable (bias between ERA5-HEAT and *in situ* data for the UTCI), as well as to assess the main effect of each independent variable and possible interactions between them. In order to achieve a balanced experimental design, the survey sample was split into equivalent data points per season, reducing the number of thermal comfort responses per season to the smallest subset, which corresponds to the summer subset with 171 responses. For that, we applied a randomization procedure to winter and fall subsets so as to fit them to equal totals with 171 responses each.

A two-way ANOVA was also performed for the urban morphological attributes of the 12 monitoring points of the survey area. As done for the season analysis, a similar procedure was adopted for balancing subsets across the considered urban morphological attributes. The most reduced sample with thermal comfort responses (221) was found for the ‘square’ subset. Survey samples from street canyon and crossroad situations were reduced to an equal size by a simple randomization procedure.

**Fig. 2:**
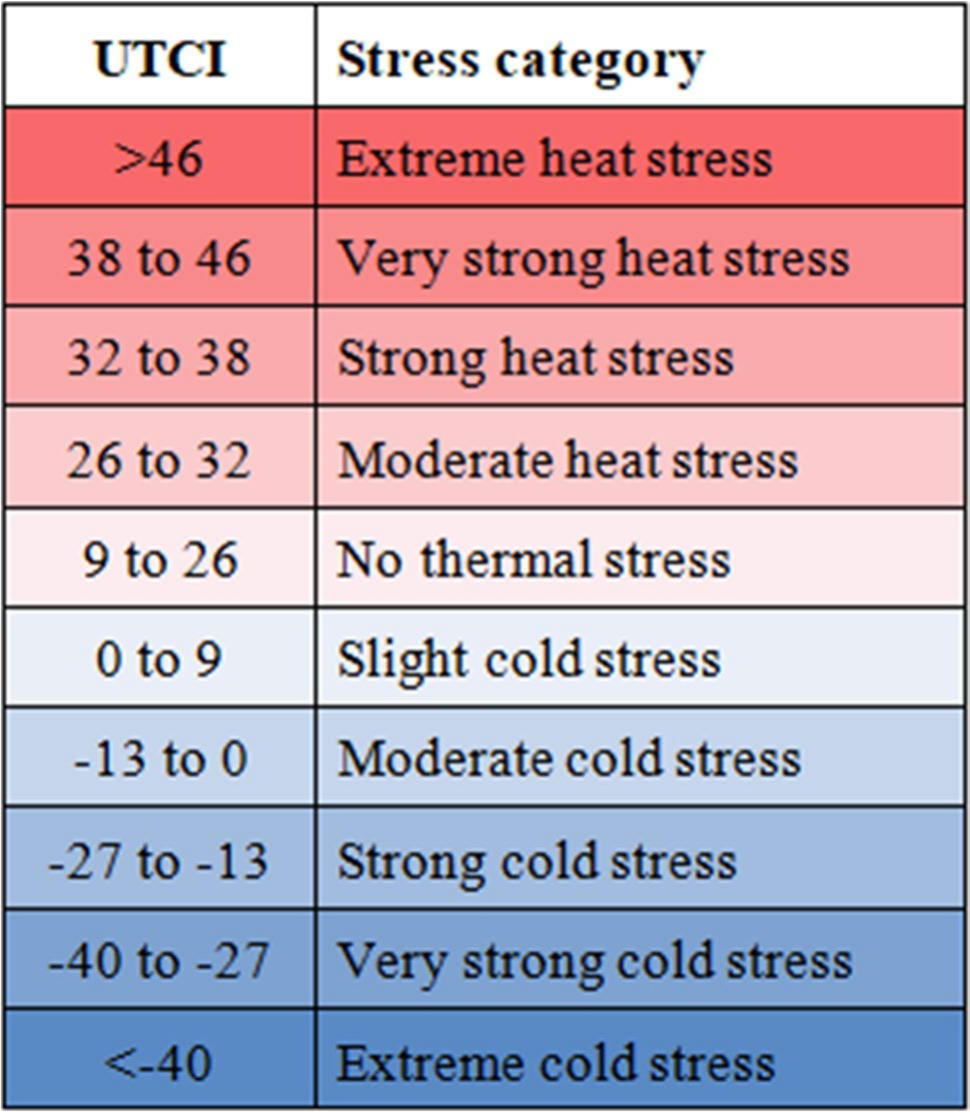
UTCI assessment scale with comfort/stress categories

As for the UTCI thermal stress categories, the conversion of UTCI values to DTS values allowed a direct comparison to the thermal sensations reported from the surveys. The comparison was performed by means of bias for both *in situ* and reanalysis datasets and by the UTCI categories as defined in the respective thermal stress assessment scale (Bröde 2021; Fig. 2).

In terms of comfort, mean thermal sensation votes (MTSVs) were calculated for binned UTCI values at each 1 °C UTCI increment. Trend lines were fitted for *in situ*-derived MTSV and reanalysis-derived MTSV data. Neutral UTCI values (where trend lines intercept the abscissa) and thresholds for the interval of ${- }{0.5\leq}\,{MTSV\leq}\,{0.5}$, here assumed as the comfort range in agreement with the definition by ISO 7730 (2005) for Class B thermal environments, were compared.

Finally, UTCI and ${T}_{MRT}$ data from the WMO weather station were compared to ERA5-HEAT reanalysis data. In the case of the field study carried out throughout different months of 2009, it can be illustrative to compare data from a local WMO weather station against reanalysis ERA5-HEAT data. For that, we used available data for January through August so as to be in agreement with the OTC survey time periods. Data were retrieved from the WMO station A807 located in Curitiba at 25°27′S, 49°14′W.

## Results

### *Comparison against *in situ* UTCI observations*

**Fig. 3:**
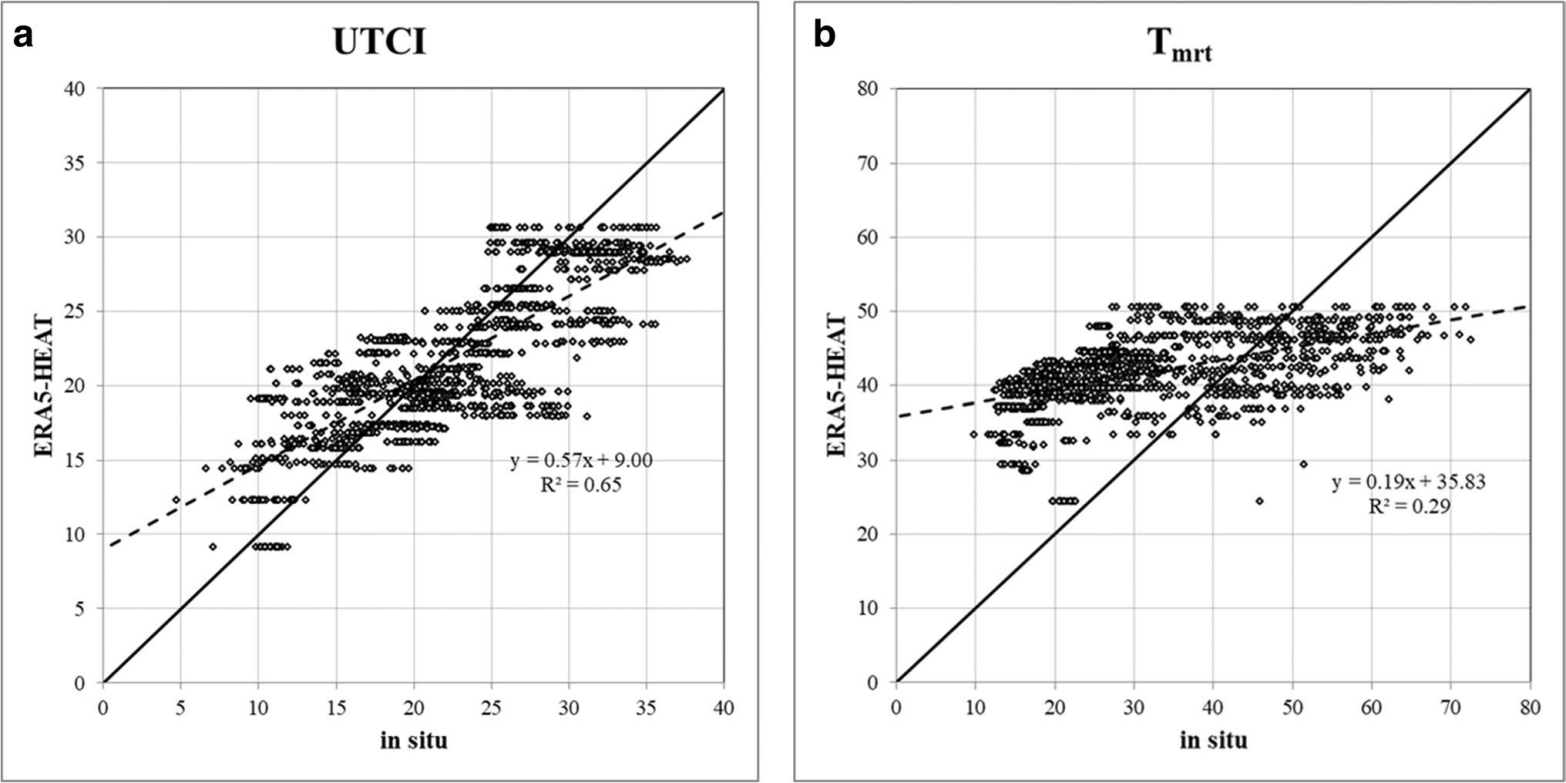
Comparison between *in situ* versus ERA5-HEAT data: **a** UTCI (°C); **b** ${T}_{MRT}$ (°C)

The comparison between measured *in situ* UTCI values and UTCI values retrieved from ERA5-HEAT is shown in Fig. 3 for the study period (January to August 2009), alongside a bulk comparison between mean radiant temperatures (${T}_{MRT}$) as calculated from *in situ* measurements of globe temperature and as extracted from ERA5-HEAT. The mean radiant temperature deserves here particular attention as this computed variable is directly affected by urban geometry resulting, for instance, from the interplay of shaded and sun-lit areas in urban canyons. Such influence was noticed in a previous publication with the same sample in Curitiba (Bröde et al. 2013). As the *in situ* dataset differs from the reanalysis dataset mainly with respect to its urban character, that influence on the ${T}_{MRT}$ is to be expected and presumably more evident than in terms of air temperature.

Linear trend lines indicate that more than 64% of ERA5-HEAT UTCI values fit the regression model with *in situ* UTCI values, whereas the percentage drops to 26% for ERA5-HEAT mean radiant temperatures.

Since *in situ* data were gathered during the exact moments of the questionnaire surveys and ERA5-HEAT data are given on an hourly basis, the dataset was gradually reduced so as to consider survey responses as close as possible to the nearest hour. For each time step, the time corresponding to each response was rounded for the hour, for half an hour and for one quarter of an hour, and only data points around each given hour were taken into account according to the adopted intervals. Table 2 shows the statistical comparison between *in situ* versus reanalysis UTCI values for the different rounding intervals.

**Table 2:** Comparisons between ERA5-HEAT versus *in situ* UTCI, in °C, for multiple evaluation scores at indicated rounding intervals. *N* indicates the number of surveys correspondingly taken

Stats	1 h	30 min	15 min	10 min
*N*	1640	812	391	282
Mean bias	- 0.81	- 0.34	- 0.11	- 0.06
Standard deviation	4.13	2.90	1.98	1.63
Pearson’s *r*	0.80	0.81	0.80	0.80
*p*-value	0.0000	0.0000	0.0254	0.1637
Maximum positive offset	10.364	10.290	10.290	9.140
Maximum negative offset	- 13.306	- 11.826	- 11.826	- 11.826

In general, it can be noticed that negligible improvements are obtained when reducing the sample to account for a narrower time interval. For rounding intervals equal to 1 h and 30 min, *p*-values are lower than the significance level of 1%, meaning that the two samples are statistically different for the complete dataset and for the half-an-hour interval. However, the series do not show statistically significant differences for shorter time intervals, with *p*-value higher than 0.01 at a 15- and 10-min rounding intervals. This suggests that hourly reanalysis data can be a reasonable proxy for missing field data when closer to the hour. As for the other evaluation metrics, the mean prediction bias and the spread (represented by the standard deviation) drop consistently for shorter rounding intervals. Pearson’s *r*-values show no substantial changes in this respect. The two offsets suggest that reanalysis data underestimate cold and heat stress. Specifically, reanalysis UTCI underestimates *in situ* UTCI at higher values (negative offset) and overestimates it for lower values. Also, the offsets reduce for shorter rounding times. From using all UTCI values to using UTCI values for the shortest rounding time of 5 min, the corresponding fluctuation of the two offsets (for under and overestimation instances) drops by nearly 3 °C UTCI, from 10.36 °C (resp. 13.31) to 9.17 °C (resp. 11.84) UTCI. Based on these results, the 1-h rounding interval is adopted in the present study.

Table 3 shows, by means of two-way ANOVA results, how the season of the year when surveys were conducted affects the difference, as represented by the mean bias, between reanalysis UTCI and *in situ* UTCI values.

**Table 3:** Two-factor ANOVA for comparisons between ERA5-HEAT versus *in situ* UTCI — seasons

Subsets	Mean bias (ERA5-HEAT minus *in situ*), in °C UTCI units	*p*-value
Fall (*N* = 171)	- 0.84	
Summer (*N* = 171)	- 3.65	
Winter (*N* = 171)	1.10	< 0.05
All seasons (*N* = 513)	- 1.13	< 0.05
Interactions (single season versus all seasons)		< 0.05

ERA5-HEAT UTCI values show a statistically significant bias relative to *in situ* UTCI values in each season. During summer, reanalysis data underestimate *in situ* data by 3.65 °C UTCI, whereas in winter, they overestimate it by 1.10 °C UTCI. In fall, the bias lies in between. The mean bias across all considered seasons points to a statistically significant underestimation of heat and cold stress by the reanalysis throughout, corroborating the results presented in Table 2. Interactions with statistical significance are found between the season-specific and the all seasons samples, suggesting that seasonality affects the goodness-of-fit of ERA5-HEAT UTCI values to *in situ* UTCI values in the total 513 survey sample.

Table 4 summarizes the results of the two-way ANOVA performed to analyse the effect of three distinct urban morphological attributes, representative of the various locations surveyed, on the bias between reanalysis and *in situ* UTCI values.

**Table 4:** Two-factor ANOVA for comparisons between ERA5-HEAT and *in situ* UTCI — urban morphology

Subsets	Mean bias (ERA5-HEAT minus *in situ*), in °C UTCI units	*p*-value
Canyon (*N* = 221)	- 0.86	
Crossroads (*N* = 221)	0.21	
Square (*N* = 221)	- 1.46	< 0.05
All urban morphological attributes (*N* = 663)	- 0.70	< 0.05
Interactions (single versus all urban morphological attributes)		0.10

Biases are negative for square and canyon settings, with the lowest bias for an intermediate condition (crossroads) and statistically significant differences between morphologies (at least between two different ones). When considering all urban morphological attributes, reanalysis and *in situ* UTCI values show statistically significant differences, though no interactions were found between the specific canyon, crossroads and square settings and the all-attributes survey sample. Thus, no inferences can be made as regards the interference of urban morphology on the goodness-of-fit of ERA5-HEAT to *in situ* UTCI values.

### *Comparison against *in situ* DTS data*

Table 5 shows evaluation scores for ERA5-HEAT versus *in situ* data in terms of the dynamic thermal sensation (DTS). Scores are presented by subsets corresponding to UTCI thermal stress categories, alongside the thermal comfort zone (TCZ) which corresponds to $18\,^\circ C\leq\,\,{UTCI\leq}\,26\,^\circ C$ (Commission for Thermal Physiology of the International Union of Physiological Sciences 2003) and the adjusted Thermal Comfort Zone for Curitiba, which is $15\;^\circ C\;\leq\;\,{UTCI \leq }\,27^\circ C\,$ (Rossi et al. 2012). The table also includes the mean thermal sensation vote (TSV) reported by respondents.

**Table 5:** Comparisons between ERA5-HEAT versus *in situ* data in terms of the dynamic thermal sensation for subsets corresponding to UTCI thermal stress ranges (Bröde et al., 2012a)

Stats (DTS)	Number	TSV	Mean *in situ* DTS	Mean ERA5-HEAT DTS	Mean bias	Standard deviation	Pearson’s *r*-value
Slight cold stress	9	- 1.0	- 1.6	- 1.1	0.6	0.2	0.59
No thermal stress	1088	- 0.2	- 0.4	- 0.3	0.1	0.4	0.63
Moderate heat stress	372	1.0	0.9	0.5	- 0.4	0.5	0.23
Strong heat stress	171	1.6	1.9	0.8	- 1.1	0.4	0.20
TCZ	659	0.1	- 0.1	- 0.2	- 0.1	0.4	0.49
TCZ adjusted	782	- 0.1	- 0.3	- 0.3	0.0	0.4	0.44

The lowest mean biases and strongest correlations are found for the ‘no thermal stress’ category, the TCZ and the adjusted TCZ. The reanalysis-*in situ* correlation of the ‘slight cold stress’ category is not considered meaningful as only 9 thermal votes fall in it. The underestimation of the reanalysis for increasing heat stress corresponds to an underestimation of the thermal sensation. This can be noticed when comparing TSV, mean *in situ* DTS and mean ERA5-HEAT DTS for the ‘moderate heat stress’ and the ‘strong heat stress’ categories.

**Fig. 4:**
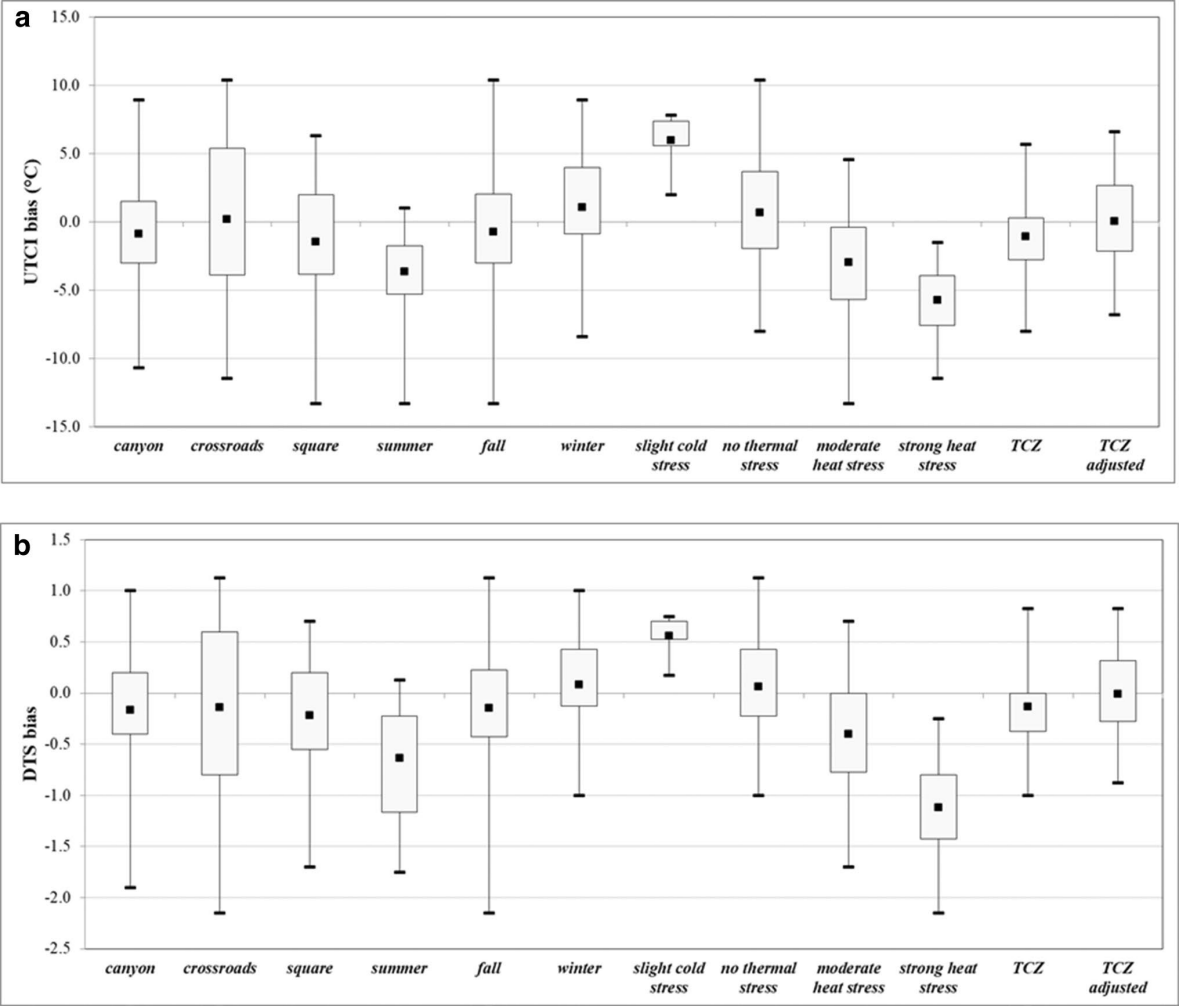
Boxplots of biases for the UTCI (**a**) and DTS (**b**) at indicated subsets. Each box represents the first (*Q*_1_ or 25th percentile) and third quartiles (*Q*_2_ or 75th percentile), the whiskers the maximum and minimum values, and the central point represents the mean value

The boxplots in Fig. 4 summarize the bias between *in situ* and ERA5-HEAT data for the UTCI and DTS, and all the subsets — seasons, urban morphological attributes, UTCI thermal stress categories — considered in the study.

For the UTCI thermal stress categories, mean biases achieve more and more negative values as heat stress increases. In the season-to-season comparison, mean biases follow similar changes, with an inversion of the bias value between summer and winter. The largest spread, represented by the difference between maximum and minimum values, is found for the fall season. For the DTS, the mean bias ranges between − 0.5 and + 0.5 in all cases but for summer and the ‘strong heat stress’ category. Such a range is considered acceptable as it is within less than one thermal vote (Rossi et al. 2012). The bias spread and relevant quartiles remain lower than a change in a thermal vote for cold (− 1 in the DTS scale) or heat (+ 1) for both the TCZ and the adjusted TCZ.

For the three urban morphology subsets, it is interesting to note that the spread of thermal votes around the mean is lowest for square settings, suggesting for these the highest goodness-of-fit of reanalysis data to *in situ* data.

### *Comparison against *in situ* MTSV data*

**Fig. 5:**
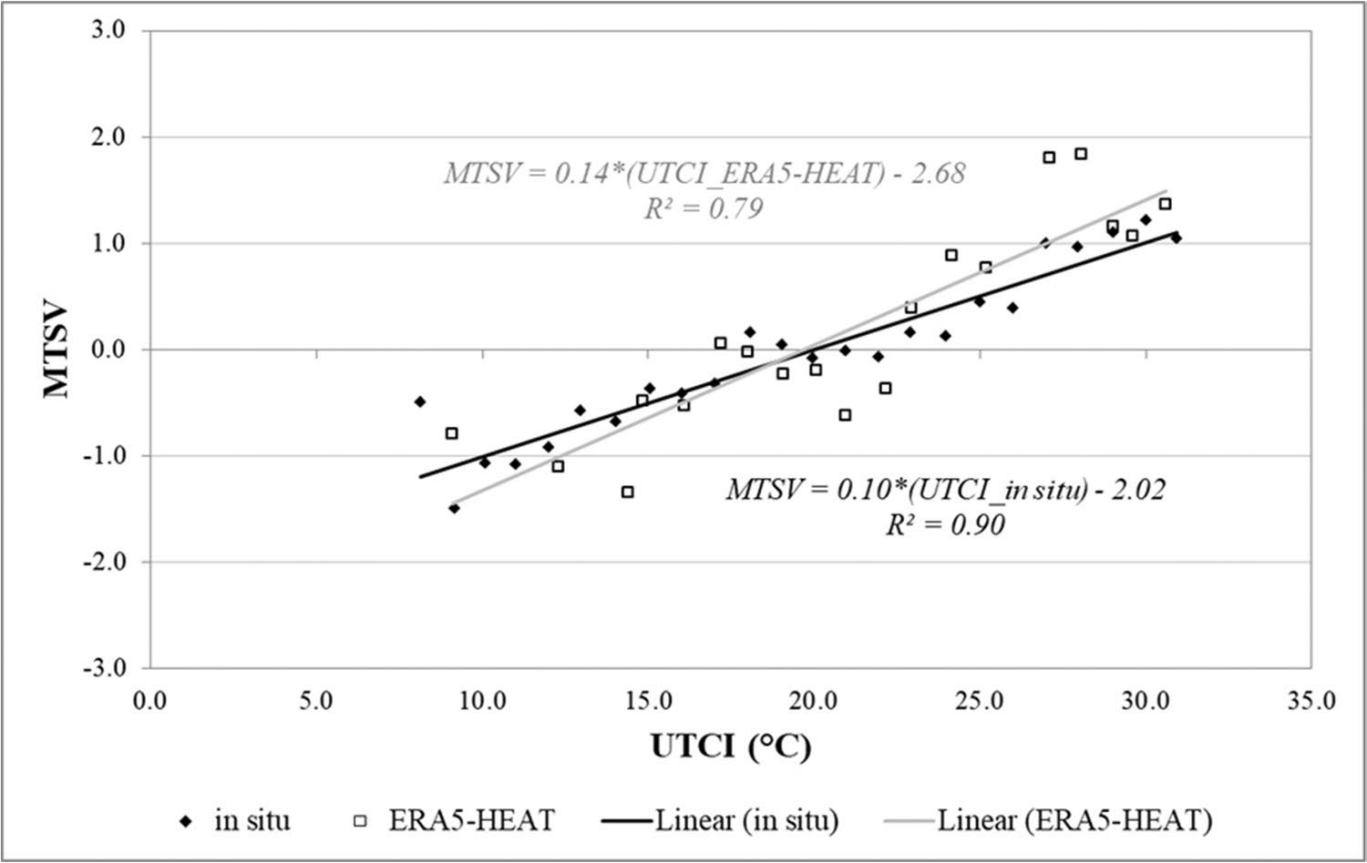
Binned thermal sensation votes (MTSV) for 1 °C increments in UTCI for the *in situ* versus ERA5-HEAT datasets

Figure 5 shows trend lines for mean thermal sensation votes (MTSVs) calculated from *in situ* and reanalysis UTCI values binned at each 1 °C UTCI increment.

The Pearson correlation coefficient is lower for reanalysis-derived MTSV data. The two trend lines have dissimilar slopes (0.10 and 0.14), which show that the sensitivity of the reported thermal votes to variations in UTCI is not the same for the two datasets. Table 6 shows, however, that the intercept with the abscissa is only slightly shifted to a lower neutral UTCI in the reanalysis data (by three decimals of a degree UTCI). Assuming the interval ${- 0.5\leq}\,{MTSV\leq}\,\,{0.5}$ as the comfort range (in agreement with the definition by ISO 7730 (2005) for Class B thermal environments), UTCI thresholds for this, i.e. the UTCI values at which ${{MTSV}}{ = }{- 0.5}$ and ${{MTSV}}{ = }{0.5}$, are reported in Table 6 for the two datasets. Thermal sensitivity is also included, informing the change in the °C UTCI for each trend line to yield a change in one MTSV.

**Table 6:** Neutral UTCI and comfort ranges for *in situ* and ERA5-HEAT data

Dataset	Trend line equation	Neutral UTCI	Lower threshold	Upper threshold	Thermal sensitivity °C/MTSV
*In situ*	MTSV = 0.10*(UTCI_*in situ*) − 2.02	20.0	15.0	24.9	9.9
ERA5-HEAT	MTSV = 0.14*(UTCI_ERA5-HEAT) − 2.68	19.7	16.0	23.4	7.3

### Comparisons to WMO station data

**Fig. 6:**
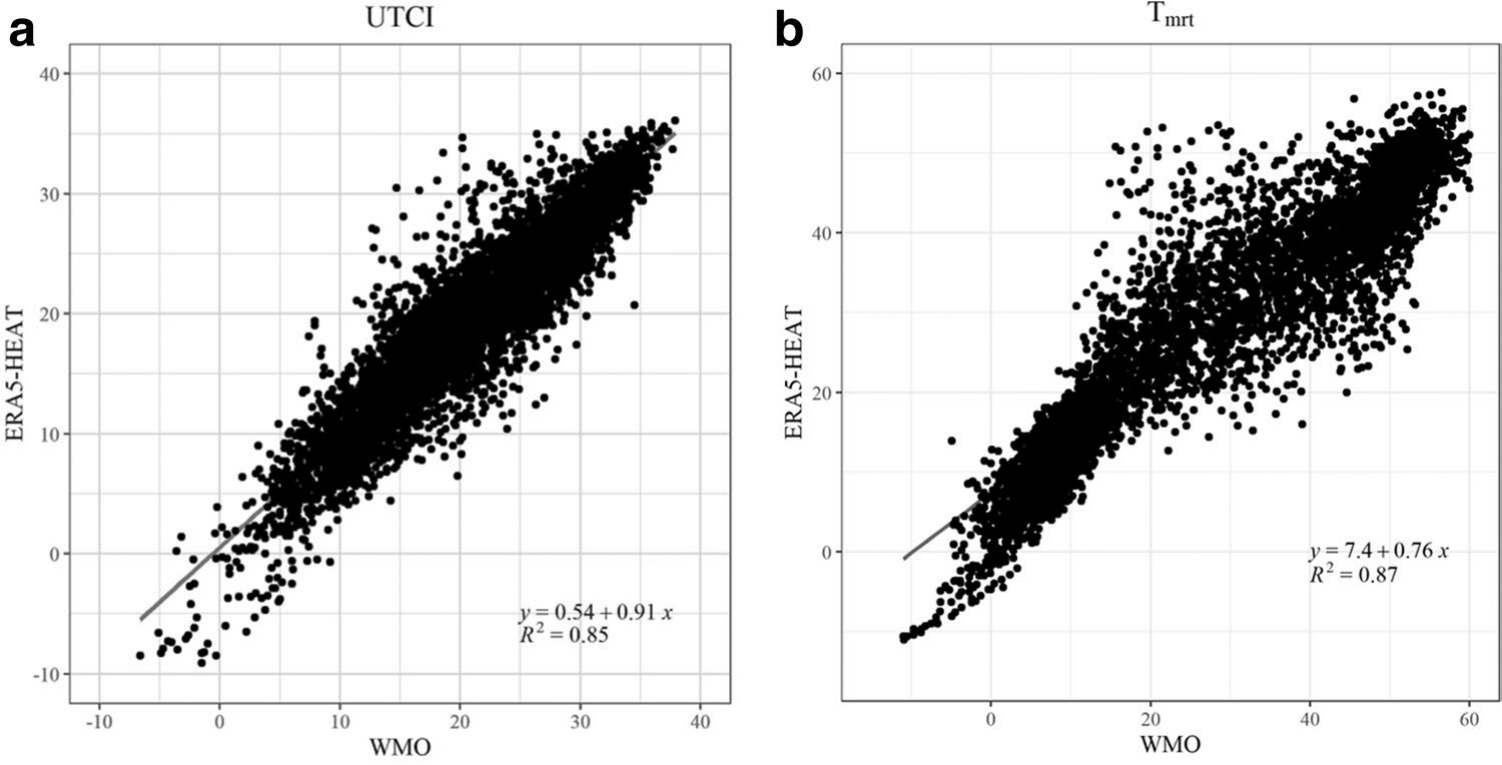
Comparisons between ERA5-HEAT versus WMO station data: **a** UTCI (°C); **b** ${T}_{MRT}$ (°C)

The WMO station dataset presents a few gaps, totalling 6066 h instead of the expected 8760 annual hours, with a 30% of missing data. For the OTC campaigns, even though only 1 h was missing (out of the 82 h during which surveys were taken), 29 thermal votes corresponding to that hour would have no reference WMO data. A comparison between hourly ERA5-HEAT and WMO station data for the January–August 2009 period is shown for the UTCI and ${T}_{MRT}$ in Fig. 6.

Pearson’s correlation coefficients are greater than 0.85 suggesting a good agreement between WMO station and reanalysis data for the UTCI and ${T}_{MRT}$.

A comparison of the reliability of hourly WMO station data against *in situ* data is given in Table 7 alongside outcomes obtained for ERA5-HEAT. Of note is the size of ERA5-HEAT data, here lower than that shown in Table 2. This is due to the aforementioned 29 data points missing in the WMO station dataset.

**Table 7:** Comparisons between *in situ* versus WMO station UTCI values, and *in situ* versus ERA5-HEAT UTCI values

Stats	ERA5-HEAT	WMO
*N*	1611	1611
Mean bias	- 0.79	2.53
Standard deviation	4.16	4.19
Pearson’s *r*	0.80	0.79
*p*-value	0.0000	0.0000
Positive maximum offset	10.364	12.349
Negative maximum offset	- 13.306	- 10.006

**Fig. 7:**
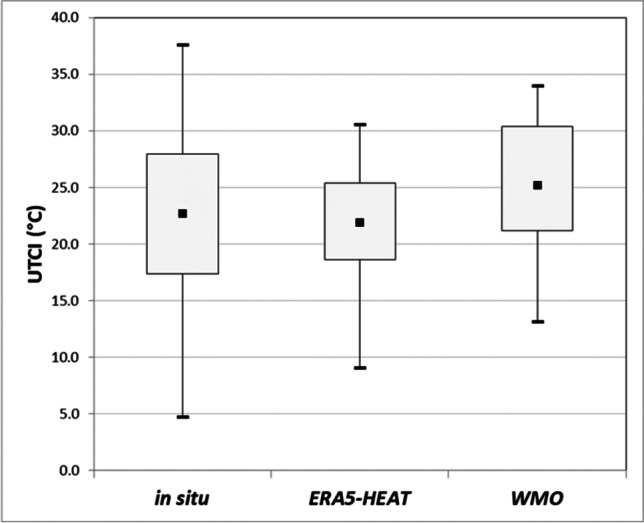
Box plot with comparisons *in situ*, ERA5-HEAT and WMO station data for the UTCI (°C) showing the first (*Q*_1_ or 25th percentile) and third quartiles (*Q*_2_ or 75th percentile), maximum and minimum values (whiskers) and the mean (central point)

The side-by-side comparison shows that evaluation metrics are in general better for reanalysis data than for the data available from the nearest meteorological station. The mean bias is generally lower, with reanalysis UTCI values slightly underestimating the *in situ* UTCI values. This can be observed also in Fig. 7, which illustrates means and spread of the UTCI for each of the three datasets.

*In situ* UTCI values show a larger spread than both reanalysis and WMO station UTCI values. This might be due to *in situ* data better capturing extreme urban effects such as hot spots, where air temperature is high and ventilation limited, or shaded spots on cold days. The way in which the UTCI and its input variables are represented in the reanalysis or sampled by *in situ* observations and by the nearby WMO station might have an impact too, and this is discussed in the next section.

## Discussion

This study provides a first evaluation of the potential usefulness and suitability of UTCI data provided by ERA5-HEAT reanalysis in the context of OTC questionnaire-based surveys in which the thermal environment is usually defined via *in situ* measurements of multiple environmental variables. Considering statistics-based evaluation metrics, ERA5-HEAT UTCI values are found to serve as a reliable backup for OTC surveys should *in situ* observations become missing and outperform WMO station data in such a purpose. Our analysis shows however that differences between reanalysis and *in situ* data, represented e.g. by the bias, do exist and must be considered. We therefore here discuss the sources of such differences.

One source is represented by the calculation of the mean radiant temperature. This was not straightforward to perform with data from the local WMO station. We used for the calculation a method that had been previously tested and compared to others when calculating the mean radiant temperature from stationary weather station data (Krüger et al. 2014). Even though the method here adopted — using air temperature, humidity, wind speed and global solar radiation measured at the meteorological station as input data in the RayMan model (Matzarakis et al. 2007) — was considered the most reliable one in that study, there was still an overestimation of the mean radiant temperature relative to *in situ* data that certainly impacted the assessment of the UTCI. Perhaps the greatest simplification in the adoption of WMO station data as a substitute for missing field data is the assumption that the SVF of the WMO station is 1, corresponding to an open field, whereas the SVFs of the surveyed points correspond to those of more obstructed urban settings, ranging for the 12 points between 0.2 and 0.55. In our case, the determination of the SVF for the 12 urban setting was done in RayMan from fisheye photos. Thus, depending on the moment of the right-here-right-now interview, the shading of direct and diffuse radiation by building structures will have affected microclimatic conditions as perceived by the respondent since the *in situ* weather station was always positioned next to the interviewees.

As for the reanalysis, the calculation of the mean radiant temperature in ERA5-HEAT is performed by setting the angle factor to 0.5 for the direct solar radiation incident on a body surface area (Di Napoli et al. 2020). This corresponds to considering the surroundings of a human body as made of a lower hemisphere (ground) and an upper hemisphere (sky) only. This assumption is valid for most applications at the macro-scale, i.e. beyond urban level (Kántor and Unger 2011), and it is consistent with the ERA5 spatial resolution of 31 km. Thus, the comparison between the mean radiant temperature from ERA5-HEAT and the mean radiant temperature from WMO station data is not straightforward and cannot be interpreted as entirely conclusive. We advise *in situ* observations, where mean radiant temperature is measured at a location closest to where OTC surveys are conducted, to be considered alongside and used as a reference.

Urban geometry can also be considered responsible for the differences between *in situ* and reanalysis UTCI. In summertime, this may cause wind-blocking effects which, combined with enhanced radiation (direct and reflected from nearby surfaces), may favour conditions of heat stress (Lau et al 2015). Shading due to surrounding buildings may also alter the thermal environment and create cool islands that, particularly in wintertime, could lead to cold stress (Zhou et al. 2020). Our analysis revealed that reanalysis data mostly resembled *in situ* data from surveyed squares. This is not surprising. In such spaces, the sky view is similar to the assumptions (angle factor equal to 0.5) made in ERA5-HEAT. We also acknowledge that the correspondence between *in situ* and reanalysis data is linked to the interplay between urban geometry, street layout and latitude. At higher latitudes, geometry will have a more pronounced effect on microclimate (Emmanuel 2021). In this respect, our analysis is limited to the subtropical climate of Curitiba and to its local latitude, as well as its urban geometry and street layout. Future research could investigate the use of ERA5-HEAT data in OTC surveys carried out in other cities across Brazil and/or across multiple climates and geographical regions, allowing comparisons to the present study.

Another aspect worth mentioning is that the bias of ERA5-HEAT UTCI to *in situ* measurements is due to the inherent uncertainties of the NWP model used to generate the reanalysis. Such uncertainties are related, for instance, to the ability of the model to represent parameters like wind speed and radiation (Pappenberger et al., 2015; Di Napoli et al. 2020). Furthermore, even at the smallest grid cell, a reanalysis is a collection of values averaged over an area. Station measurements, conversely, are values collected at one specific point. The difference between reanalysis and measurements increases as the grid cell size increases, the terrain becomes complex and its surface changes roughness and type, as it happens for cities. This further supports the importance to compare reanalysis data to *in situ* observations and apply bias corrections where needed. Moreover, downscaling methods may be used to process reanalysis data and refine their climate information at more local scales. One of these methods is statistical downscaling which uses statistics-based techniques to correlate reanalysis data to local observations. Statistical downscaling has been successfully applied, for instance, to transform large-scale climatic variables from the NCEP/NCAR reanalysis into local-scale climatic variables for the urban area of Hong Kong (Cheung and Hart 2014). The downscaled variables were then used to calculate the UTCI and the probability distribution of its thermal stress categories in Hong Kong over the historical period 1979–2000. Downscaling methods have also been used for predicting the UTCI in the near and remote future both in Europe and Asia (Brecht et al. 2020; Cheung and Hart 2014, Di Napoli et al., 2021b). As the UTCI is predicted to reach ‘extreme heat stress’ levels in Brazil at the end of the twenty-first century (de Souza Hacon et al. 2019), future research efforts could aim at downscaling the UTCI at multiple Brazilian cities. This would provide quantitative information on the urban environment, and the impacts of climate change on it thus can be used as a guide for local urban planning.

## Conclusions

In this study, the feasibility of using UTCI data from the ERA5-HEAT reanalysis dataset as a proxy for onsite measurements of the UTCI is assessed for the first time. The assessment is made in the context of outdoor thermal comfort and is based on a sample of 1640 surveys carried out between January and August 2009 in Curitiba, a city in southern Brazil. The survey consists of questionnaires on thermal perception that were given to local pedestrians and were linked to microclimatic conditions measured *in situ* by portable weather stations. From these, *in situ* UTCI values were calculated and compared to UTCI values retrieved from ERA5-HEAT for the same location and period. The comparison was performed by means of statistical evaluation metrics and includes measurements from a nearby WMO station (A807) which was used as an additional reference.

Relevant findings can be summarized as follows:

- As ERA5-HEAT data are given on an hourly basis, the mean bias and standard deviation of reanalysis UTCI values to *in situ* UTCI values can be greatly reduced when accounting for survey responses close to the hour
- Reanalysis data can be used (a) in the fall season when the bias reduces but the consistency with *in situ* data diminished due to a non-negligible spread, or (b) in the winter season when the spread is less but there is a slight overestimation of the UTCI
- Replacement of missing *in situ* data by reanalysis data can be considered when thermal conditions in the UTCI assessment scale are neutral or within the TCZ, as bias and spread are reduced
- Reanalysis data outperformed data from the stationary WMO station as they present less bias, less spread in terms of standard deviation and higher correlation to *in situ* data, thus serving as reliable backup reference for OTC surveys.

The observations made above can be useful and guide researchers in the case of flawed or missing field data and in the possibility of replacing them with ERA5-HEAT reanalysis data. As the outcomes of the study are limited to the location of Curitiba, Brazil, future research could verify the extent of the statements we put forward in this study by testing the replacement of *in situ* data with reanalysis data in other latitudes and climate regions across the world.

Provided that the procedures described here yield similar results across various locations, another venue worth investigating would be to compare reported thermal perception by taking ERA5-HEAT data as standard weather data. This would allow differences due to station equipment and monitoring protocols to be greatly reduced, and specific studies crossing OTC and sociocultural, regional and acclimatization effects to be understood on a meteorologically consistent ground.

## Data Availability

The datasets generated during and/or analysed during the current study are not publicly available due to undisclosed reasons but can be made available from the corresponding author on reasonable request. The ERA5-HEAT data used in this study can be extracted from the ‘Thermal comfort indices derived from ERA5 reanalysis’ which is freely available at the Copernicus Climate Data Store with the identifier 10.24381/cds.553b7518.
